# Elevated thyroid autoantibodies as risk factors for metabolic dysfunction-associated fatty liver disease in type 2 diabetes mellitus

**DOI:** 10.3389/fendo.2024.1478818

**Published:** 2024-12-05

**Authors:** Wenchang Wu, Ziyi Yang, Owen Li, Lulu Gan, Yue Gao, Cheng Xiang, Ling Li, Yimin Yan

**Affiliations:** ^1^ Medical College, Wuhan University of Science and Technology, Wuhan, China; ^2^ Department of Endocrinology, Xiaogan Hospital Affiliated to Wuhan University of Science and Technology, The Central Hospital of Xiaogan, Xiaogan, Hubei, China

**Keywords:** type 2 diabetes mellitus, metabolic dysfunction-associated fatty liver disease, thyroid autoantibodies, thyroid peroxidase antibody, thyroglobulin antibody

## Abstract

**Objective:**

This study aims to explore the relationship between thyroid peroxidase antibody (TPOAb) and thyroglobulin antibody (TgAb) levels and metabolic dysfunction-associated fatty liver disease (MAFLD) in patients with type 2 diabetes mellitus (T2DM), providing a theoretical basis for MAFLD prevention and treatment.

**Methods:**

From June 2020 to May 2023, 534 T2DM patients were selected from the Endocrinology Department of Xiangyang Hospital affiliated with Wuhan University of Science and Technology. After applying exclusion criteria, 432 subjects were included. Based on abdominal ultrasound and MAFLD diagnostic criteria, subjects were divided into non-MAFLD (n=163) and MAFLD (n=260) groups. Differences in various indicators between the two groups were compared. Correlation analysis assessed the relationship between TPOAb, TgAb, and other indicators, and the prevalence of MAFLD was analyzed under different thyroid function and antibody levels. Multivariate logistic regression identified risk factors for MAFLD in T2DM patients. According to the FIB-4 index, the group with MAFLD was divided into low-risk (FIB-4< 1.30, n=150), medium-risk (1.30≤FIB-4 ≤ 2.67, n=100), and high-risk liver fibrosis groups (FIB-4 > 2.67, n=10). Differences in thyroid function and autoantibody levels among the three groups were compared.

**Results:**

Compared to non-MAFLD patients, 73.46% of MAFLD patients were overweight or obese, were younger, and had a shorter duration of diabetes. Under normal thyroid function, MAFLD patients had higher levels of TSH, TgAb, and TPOAb (*P*<0.05). The prevalence of TgAb+, TPOAb+, and TgAb/TPOAb+ was significantly higher at 21.9%, 22.1%, and 29.6%, respectively, with higher prevalence in females. Spearman’s correlation showed a positive correlation between TgAb, TPOAb, and AST, and between TPOAb and FINS. MAFLD prevalence varied among quartiles of TSH, TPOAb, and TgAb levels, with significant differences in TPOAb and TgAb components (*P*<0.05). MAFLD prevalence was positively correlated with TgAb and TPOAb levels. Thyroid autoantibody-positive patients had a significantly higher MAFLD prevalence (*P*=0.010) at 71.96%. Multivariate logistic analysis found elevated TSH and TPOAb levels as risk factors for MAFLD in T2DM patients [(OR 1.441, 95% CI: 1.213-1.712, *P*<0.001), (OR 1.005, 95% CI: 1.000-1.010, *P*=0.040)]. Medium-risk liver fibrosis patients had higher TgAb and TPOAb levels than low-risk and high-risk groups [TgAb: 1.04(0.59,2.83) vs 1.54(0.76,7.35) vs 0.55(0.27,1.32), P=0.035; TPOAb: 1.0(0.29,3.83) vs 2.42(0.5,23.08) vs 0.17(0.09,2.71), *P*=0.002]. Further comparisons revealed a significant difference in TgAb levels between the medium-risk and high-risk groups (*P* = 0.048). Additionally, significant differences in TPOAb levels were observed between the low-risk and medium-risk groups and between the medium-risk and high-risk groups (*P* = 0.016,*P* = 0.014).

**Conclusion:**

In T2DM patients with MAFLD, elevated TSH, TgAb, and TPOAb levels are observed under normal thyroid function. Elevated TSH and TPOAb levels are risk factors for MAFLD in T2DM patients. TgAb and TPOAb levels vary among liver fibrosis risk groups, showing an inverted “V” pattern, suggesting a role in MAFLD progression to liver fibrosis.

## 1 Introduction

Metabolic dysfunction-associated fatty liver disease (MAFLD) is a prevalent, chronic, and progressive liver disease with global implications, potentially leading to cirrhosis and liver cancer (1). In 2020, an expert consensus redefined non-alcoholic fatty liver disease as MAFLD, highlighting its distinctiveness as a complex multisystem disorder (2). Studies indicate that with the rising prevalence of obesity and diabetes, the incidence of MAFLD is expected to increase (3). The pathophysiology of MAFLD is complex and multifactorial, with innate immune mechanisms and inflammatory responses being crucial contributors (4, 5).

Thyroid dysfunction is recognized as a risk factor for MAFLD. Thyroid hormones influence adipocyte differentiation, adipose tissue inflammation, and hepatic lipid metabolism. Recent research has demonstrated a close relationship between thyroid autoimmunity and metabolic disturbances (6). Autoimmune thyroid disease can result in thyroid dysfunction, which is closely associated with the onset of MAFLD. Immune and chronic inflammatory responses play pivotal roles in the pathogenesis of both autoimmune thyroid disease and MAFLD. However, among type 2 diabetes mellitus (T2DM) patients with normal thyroid function, few studies have examined the direct correlation between autoimmune thyroid disease and MAFLD (7).

The mechanisms underlying the relationship between thyroid peroxidase antibodies (TPOAb), thyroglobulin antibodies (TgAb), and MAFLD in T2DM patients involve immunoregulatory, metabolic, and hormonal factors. Immune responses—including interactions between thyroid and hepatic immunity and alterations in regulatory T cells—play a significant role. Metabolic factors such as lipid metabolism disorders and insulin resistance affect thyroid cells and function. Hormonal mechanisms, including disruptions of the hypothalamic-pituitary-thyroid axis and the influence of hormones like leptin and adiponectin, also contribute.

Studying the association between thyroid autoantibodies and MAFLD in T2DM patients is important for several reasons (8). Firstly, diabetes patients have a higher prevalence of metabolic disorders, and MAFLD is closely related to metabolic dysregulation. Investigating this association can enhance our understanding of the specific pathophysiological mechanisms underlying the co-occurrence of these conditions within the context of altered metabolism. Secondly, diabetes often leads to multiple complications, and understanding this relationship may have significant implications for the management and prognosis of diabetes patients. It could help identify new risk factors and therapeutic targets to improve comprehensive care and reduce the risk of associated comorbidities. Thirdly, diabetes patients may have unique immune and hormonal profiles that interact differently with thyroid autoantibodies and MAFLD compared to the general population. Understanding these specific relationships can provide valuable insights into the complex interplay between different diseases and contribute to the development of more personalized and targeted treatment strategies for this group of patients.

Therefore, this study aims to investigate the correlation between TgAb and TPOAb levels and MAFLD in T2DM patients with normal thyroid function.

## 2 Patients and methods

### 2.1 Patients

A total of 534 type 2 diabetes mellitus (T2DM) patients hospitalized in the Endocrinology Department of Xiaogan Hospital affiliated with Wuhan University of Science and Technology and who underwent abdominal ultrasound examinations from June 2020 to May 2023 were selected for this study.

### 2.2 Inclusion and exclusion criteria

T2DM was diagnosed according to the 1999 World Health Organization (WHO) criteria (9): ① Diabetes symptoms plus random blood glucose ≥11.0 mmol/L; ② Fasting venous blood glucose ≥7.0 mmol/L; ③ OGTT 2-hour blood glucose ≥11.0 mmol/L. Diagnosis required meeting any one of these criteria.

Exclusion criteria were as follows: (1) Age<18 years; (2) History of hyperthyroidism, hypothyroidism, thyroid tumors, thyroid surgery, neck trauma, or use of medications affecting thyroid function; (3) Factors contributing to secondary hepatic fat accumulation, such as alcoholism, hepatotoxic drugs, autoimmune hepatitis, genetic diseases, or other liver diseases; (4) Severe malnutrition, mental illness, malignancy, severe infection, or infectious diseases; (5) Type 1 diabetes, gestational diabetes, specific types of diabetes, diabetic ketoacidosis; (6) Missing key data.

After applying the exclusion criteria, 432 patients were included in the study. The diagnosis of MAFLD was based on abdominal ultrasound results and the International Expert Consensus on the New Definition of Metabolic Dysfunction-Associated Fatty Liver Disease (5). Of the 432 patients, 260 (approximately 61.5%) were diagnosed with MAFLD. MAFLD patients were further categorized into low-risk (FIB-4<1.30, n=150), medium-risk (1.30 ≤ FIB-4 ≤ 2.67, n=100), and high-risk liver fibrosis groups (FIB-4 >2.67, n=10). The study protocol was approved by the Ethics Committee of Xiangyang Central Hospital, Wuhan University of Science and Technology. All subjects provided written informed consent.

### 2.3 Data collection

The patients’ gender, age, medical history, height, weight, blood pressure, and other personal information were obtained from the hospital information system. The following laboratory indicators were collected:High-sensitivity C-reactive protein (HsCRP), Lymphocyte count (Lym), Alanine aminotransferase (ALT), Aspartate transaminase (AST), Albumin (ALB),γ-Glutamyl transferase (GGT), Triglyceride (TG), Total cholesterol (TC), High-density lipoprotein cholesterol (HDL-C), Low-density lipoprotein cholesterol (LDL-C), Fasting blood glucose (FBG), Fasting insulin (FINS), Glycosylated hemoglobin (HbA1c), Free triiodothyronine (FT3), Free thyroxine (FT4), Thyroid stimulating hormone (TSH), Thyroid peroxidase antibody (TPOAb), Thyroglobulin antibody (TgAb).

The reference ranges for normal thyroid function indicators in our hospital are as follows: TSH (0.35–4.94 uIU/mL), FT3 (2.43–6.01 pmol/L), FT4 (9.01–19.05 pmol/L), TgAb (0–4.11 U/mL), and TPOAb (0–5.61 U/mL). Positive TPOAb (serum level > 5.61 U/mL) and/or positive TgAb (serum level > 4.11 U/mL) were considered indicative of thyroid autoimmunity. Patients positive for TgAb and/or TPOAb were denoted as TgAb/TPOAb+.

N/L represents the ratio of neutrophil count (Neu) to lymphocyte count. M/L represents the ratio of monocyte count (Mon) to lymphocyte count. The following formulas were used to calculate the respective indices:

Homeostasis model assessment of insulin resistance (HOMA-IR): $\text{HOMA}-\text{IR}=\frac{\text{FBG}\left(\text{mmol}/\text{L}\right)\times \text{FINS}\left(\text{µU}/\text{mL}\right)}{22.5}$ .

Triglyceride glucose index (TyG): $\text{TyG}=\text{In}\left(\frac{\text{TG}\left(\text{mg}/\text{dl}\right)\times \text{FBG}\left(\text{mg}/\text{dl}\right)}{2}\right)$ .

Fibrosis-4 index (FIB-4): $\text{FIB}-4=\frac{\text{Age}\left(\text{years}\right)\times \text{AST}\left(\text{U}/\text{L}\right)}{\text{PLT}\left(10^{9}/\text{L}\right)\times \sqrt{\text{ALT}\left(\text{U}/\text{L}\right)}}$ .

## 3 Statistical analysis

Data processing and analysis were conducted using SPSS 27.0. Normally distributed continuous variables are presented as mean ± standard deviation, and comparisons between two groups were performed using an independent samples t-test. Due to the non-normal distribution of the data, comparisons among the three groups were performed using the Kruskal-Wallis H test. If the test indicated statistical significance, pairwise comparisons were conducted using the Mann-Whitney U test, with P-values adjusted using the Bonferroni correction. Spearman’s correlation analysis was used to assess the relationships between variables. The prevalence of MAFLD under different thyroid function levels and immune statuses was analyzed. Binary logistic regression analysis was performed to evaluate the impact of thyroid autoantibody levels on the prevalence of MAFLD. Statistical significance was set at *P<* 0.05.

## 4 Results

### 4.1 Clinical characteristics of the two groups

**Figure 1:**
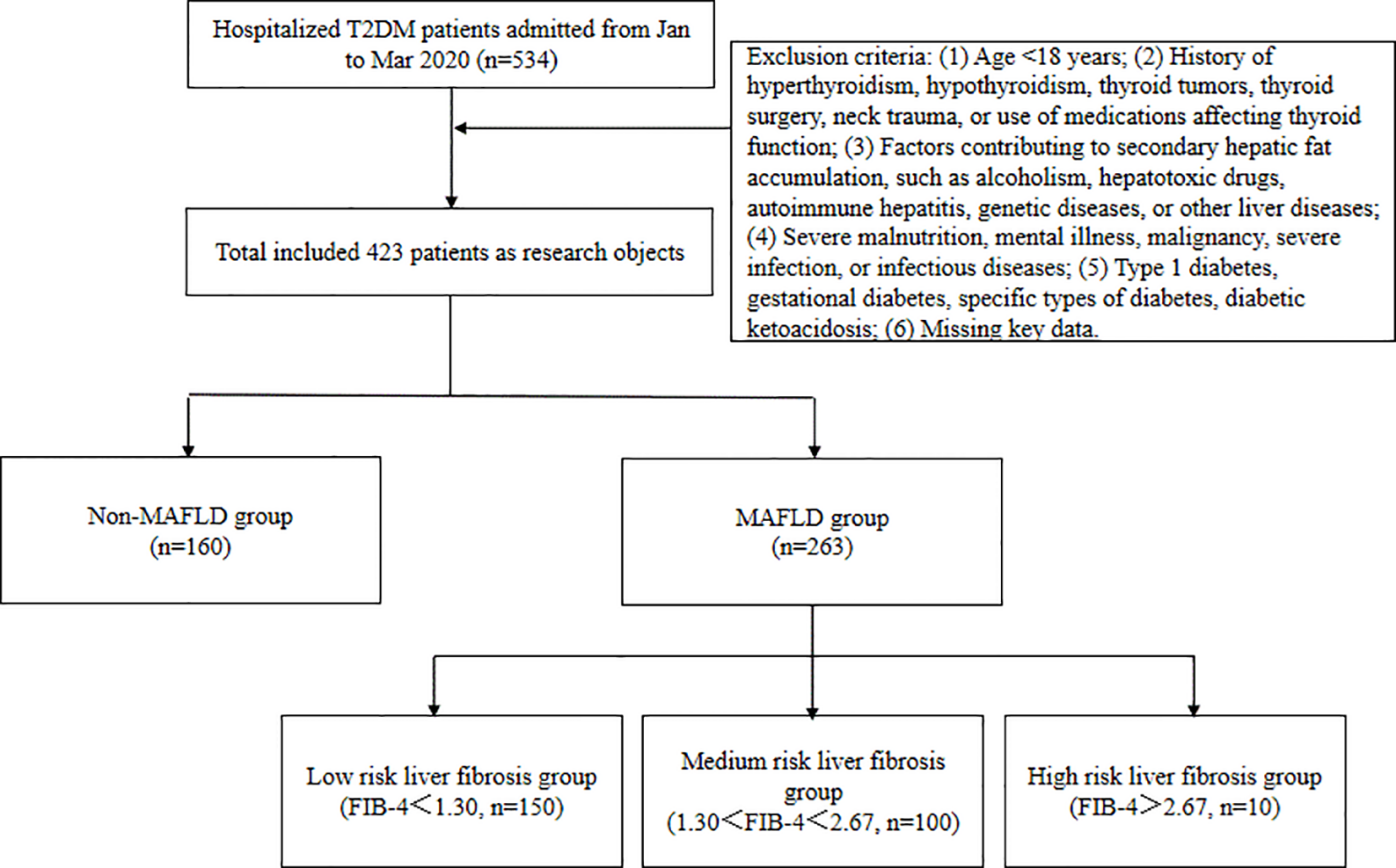
Flowchart of subject selection.

The subject selection process is illustrated in **Figure 1**, and the clinical characteristics of the two groups are summarized in **Table 1**. A total of 432 type 2 diabetes mellitus (T2DM) patients were included in the study, with an average age of 54.65 ± 12.14 years; 60.3% were male and 39.7% were female. Among these, 260 patients (61.5%) were diagnosed with MAFLD. The MAFLD group had significantly higher BMI levels and a higher proportion of females compared to the non-MAFLD group. However, the MAFLD group was significantly younger and had a shorter duration of diabetes. Additionally, approximately 73.46% of the overweight and obese individuals (BMI ≥ 24 kg/m²) were in the MAFLD group, significantly higher than in the non-MAFLD group.

**Table 1:** Clinical characteristics of the two study groups.

Variables	non-MAFLD (n=163)	MAFLD (n=260)	χ2/Z/F	*P*
Women (n, %)	52 (31.9%)	116 (44.6%)	6.764	0.009
Age (year)	57.4 ± 11.36	52.92 ± 12.32	14.100	<0.001
Duration of diabetes (years)	7 (1.5,12)	3 (0.3,9)	-3.954	<0.001
BMI (kg/m2)	23.83 (21.45,25.61)	25.69 (23.84,28.05)	-7.088	<0.001
<24kg/m2	85 (52.15%)	69 (26.54%)		
≥24kg/m2	78 (47.85%)	191 (73.46%)		
Lym (×10^9/L)	1.66 (1.37,2.08)	1.91 (1.53,2.28)	-3.557	<0.001
N/L	1.82 (1.41,2.45)	1.74 (1.29,2.24)	-1.987	0.047
M/L	0.2 (0.17,0.25)	0.18 (0.14,0.23)	-3.151	0.002
HsCRP (mg/L)	1.51 (1.12,2.74)	2.16 (1.52,3.83)	-4.215	<0.001
ALT (U/L)	14 (10,24)	20 (14,35)	-5.667	<0.001
AST (U/L)	18 (15,22)	19.5 (15.25,27)	-3.201	0.001
GGT (U/L)	19 (14,34)	28 (20,45.75)	-5.125	<0.001
TC(mmol/L)	4.24 (3.45,4.94)	4.57 (3.94,5.26)	-3.414	<0.001
TG (mmol/L)	1.14 (0.84,1.67)	1.64 (1.19,2.87)	-7.073	<0.001
HDL-C (mmol/L)	1.14 (0.94,1.4)	1.01 (0.8,1.23)	-4.319	<0.001
LDL-C (mmol/L)	2.25 ± 0.77	2.49 ± 0.78	-2.871	0.004
TG/HDL-C	0.94 (0.68,1.62)	1.72 (1.04,3.08)	-6.898	<0.001
HA1bc (%)	8.2 (6.6,10.1)	9.01 (7.2,11)	-2.219	0.027
FBG (mmol/L)	7.66 (5.9,10.68)	8.06 (6.52,10.57)	-1.427	0.154
FINS (mmol/L)	5.2 (3.53,8.4)	7.39 (4.88,11.18)	-4.539	<0.001
HOMA-IR	1.91 (1.03,3.38)	2.7 (1.73,4.23)	-4.546	<0.001
TyG	7.32 (6.85,7.79)	7.81 (7.31,8.22)	-6.332	<0.001

The MAFLD group showed significantly higher levels of lymphocytes (Lym), high-sensitivity C-reactive protein (HsCRP), alanine aminotransferase (ALT), aspartate transaminase (AST), γ-glutamyl transferase (GGT), total cholesterol (TC), triglycerides (TG), low-density lipoprotein cholesterol (LDL-C), TG/HDL-C ratio, glycated hemoglobin (HbA1c), fasting insulin (FINS), HOMA-IR, and triglyceride-glucose index (TyG) (*P*< 0.05). Although the fasting blood glucose (FBG) level was higher in the MAFLD group [7.66 (5.9, 10.68) vs 8.06 (6.52, 10.57)], the difference was not statistically significant (*P* = 0.154). In contrast, the levels of high-density lipoprotein cholesterol (HDL-C), neutrophil to lymphocyte ratio (N/L), and monocyte to lymphocyte ratio (M/L) were significantly lower in the MAFLD group (*P<* 0.05).

### 4.2 Thyroid function indicators of the two groups

The thyroid function indicators for both groups are summarized in **Table 2**. All subjects had FT3 and FT4 levels within the normal range. The MAFLD group had lower levels of FT3 [4.09 (3.65, 4.56) vs 4.08 (3.7, 4.47), *P*=0.747] and FT4 [12.93 (11.98, 13.96) vs 12.58 (11.4, 13.78), *P*=0.053] compared to the non-MAFLD group, though these differences were not statistically significant. Conversely, the MAFLD group showed significantly higher levels of TSH [1.95 (1.33, 2.91) vs 2.98 (1.92, 4.05)], TgAb [0.95 (0.46, 1.69) vs 1.07 (0.65, 3.62)], and TPOAb [0.82 (0.25, 3.43) vs 2.13 (0.37, 4.83)] compared to the non-MAFLD group (*P*<0.05). Additionally, the prevalence rates of TgAb+, TPOAb+, and TgAb/TPOAb+ were significantly higher in the MAFLD group (*P*<0.05). Furthermore, within the MAFLD group, the prevalence of TgAb/TPOAb+ was 24.31% in males and 36.21% in females, though this difference was not statistically significant (χ2 = 2.352, *P*=0.152).

**Table 2:** Thyroid function indicators of the two study groups.

Variables	non-MAFLD (n=163)	MAFLD (n=260)	χ2/Z/F	*P*
FT3 (pmol/L)	4.09 (3.65,4.56)	4.08 (3.70,4.47)	-0.322	0.747
FT4 (pmol/L)	12.93 (11.98,13.96)	12.58 (11.4,13.78)	-1.933	0.053
TSH (uIU/mL)	1.95 (1.33,2.91)	2.98 (1.92,4.05)	-5.728	<0.001
TgAb (U/mL)	0.95 (0.46,1.69)	1.07 (0.65,3.62)	-2.568	0.010
TPOAb (U/mL)	0.82 (0.25,3.43)	2.13 (0.37,4.83)	-3.787	<0.001
TgAb +	20 (12.3%)	57 (21.9%)	6.270	0.012
TPOAb +	23 (14.1%)	58 (22.1%)	4.348	0.037
TgAb/TPOAb+	30 (11.4%)	77 (29.6%)	6.663	0.010

### 4.3 Correlation analysis between TPOAb, TgAb, and various indicators

**Figure 2:**
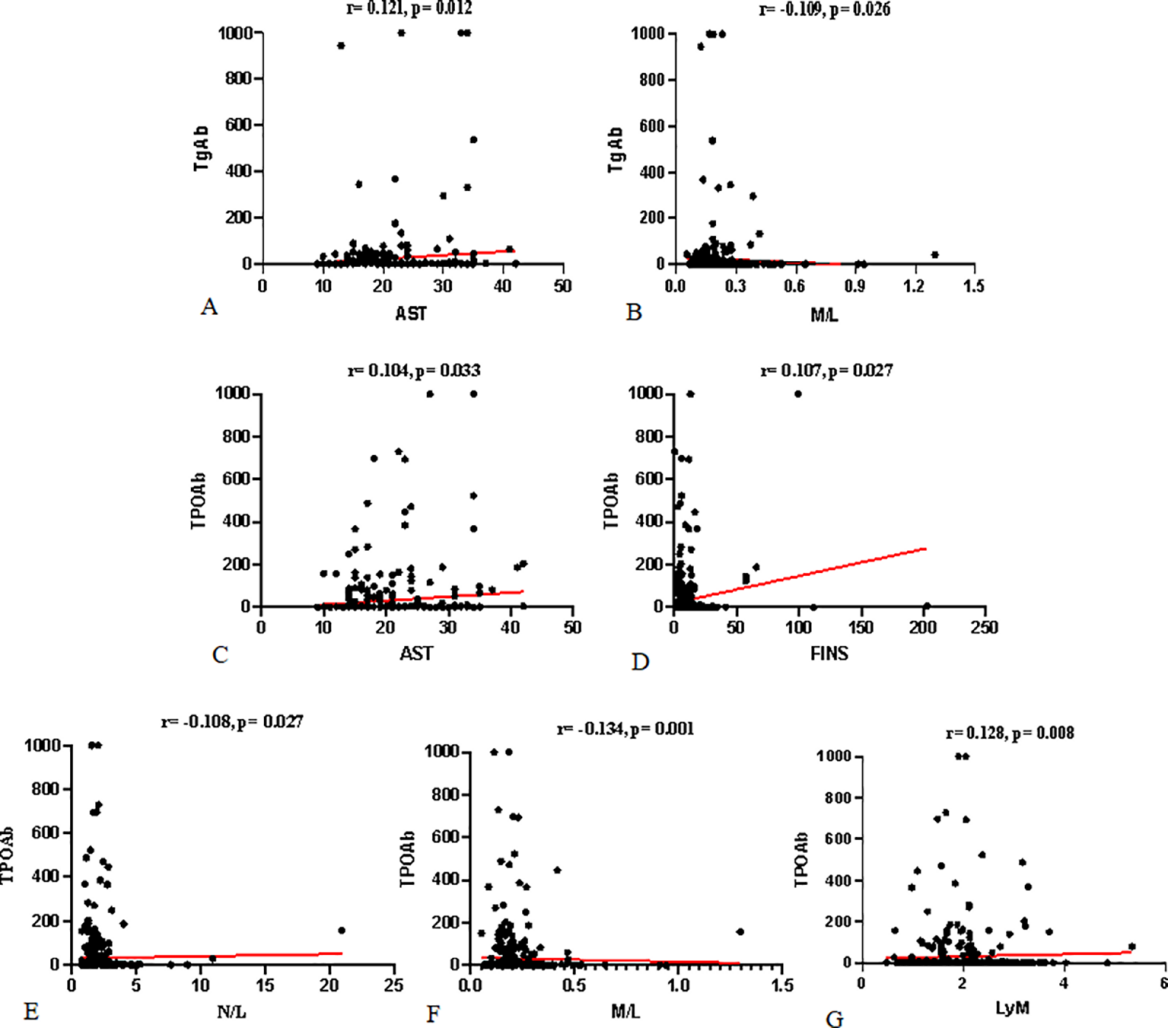
Correlation between TgAb, TPOAb, and Various Indicators: **(A, B)** show the correlations between TgAb and AST and M/L. **(C–G)** show the correlations between TPOAb and AST, FINS, N/L, M/L, and Lym, respectively.

The correlation analysis between TPOAb, TgAb, and various indicators is presented in **Table 3** and **Figure 2**. TgAb showed a significant positive correlation with AST (r = 0.121; *P* = 0.012) and a significant negative correlation with the monocyte-to-lymphocyte ratio (M/L) (r = -0.109; *P* = 0.026). TPOAb demonstrated significant positive correlations with AST, fasting insulin (FINS), and lymphocyte count (Lym) (r = 0.104, 0.107, 0.128; *P* = 0.033, 0.027, 0.008), and significant negative correlations with the neutrophil-to-lymphocyte ratio (N/L) and M/L (r = -0.108, -0.156; *P* = 0.027, 0.001).

**Table 3:** Correlation analysis between TPOAb, TgAb, and various indicators.

Variables	TgAb		TPOAb	
	r	*P*	r	*P*
BMI (kg/m2)	0.059	0.226	0.071	0.146
Lym (×10^9/L)	0.077	0.116	0.128	0.008
N/L	-0.091	0.060	-0.108	0.027
M/L	-0.109	0.026	-0.156	0.001
ALT (U/L)	0.052	0.290	0.062	0.201
AST (U/L)	0.121	0.012	0.104	0.033
GGT (U/L)	0.045	0.359	0.051	0.293
TC (mmol/L)	0.078	0.110	0.090	0.064
TG (mmol/L)	0.008	0.866	0.008	0.872
HDL-C (mmol/L)	-0.076	0.117	-0.051	0.300
LDL-C (mmol/L)	0.050	0.308	0.092	0.059
TG/HDL-C	0.023	0.633	0.016	0.737
HsCRP (mg/L)	0.089	0.069	-0.005	0.920
HAlbc (%)	0.017	0.725	-0.017	0.722
FINS (mmol/L)	0.077	0.114	0.107	0.027
HOMA-IR	0.074	0.127	0.077	0.116
TyG	0.008	0.864	-0.005	0.915

### 4.4 Binary logistic regression analysis

The impact of thyroid function and thyroid autoimmunity on the prevalence of MAFLD is summarized in **Table 4**. Model 1, adjusted for confounding variables, indicates that higher TSH levels are associated with an increased risk of MAFLD in type 2 diabetes patients (OR 1.486, 95% CI: 1.259-1.754, *P*<0.001). Model 2, which includes further adjustments, shows that elevated levels of both TSH and TPOAb are linked to a higher risk of MAFLD in type 2 diabetes patients [(OR 1.441, 95% CI: 1.213-1.712, *P*<0.001), (OR 1.005, 95% CI: 1.000-1.010, *P*=0.040)]. These findings suggest that increased TSH and TPOAb levels are significant risk factors for the development of MAFLD in T2DM patients.

**Table 4:** Logistic regression analysis of thyroid function and autoimmune markers with MAFLD.

Variables	Model 1			Model 2		
	OR	95%CI	*p*	OR	95%CI	*P*
FT3 (pmol/L)	1.013	0.688-1.491	0.948	0.871	0.566-1.340	0.529
FT4 (pmol/L)	0.984	0.862-1.123	0.811	1.004	0.873-1.156	0.954
TSH (uIU/mL)	1.486	1.259-1.754	<0.001	1.441	1.213-1.712	<0.001
TgAb (U/mL)	1.000	0.997-1.004	0.763	1.001	0.998-1.004	0.541
TPOAb (U/mL)	1.003	1.000-1.007	0.067	1.005	1.000-1.010	0.040

Model 1: Adjusted for gender, age, BMI, duration of diabetes.

Model 2: Further adjusted for ALT, AST, TC, HDL-C, LDL-C, HsCRP, Lym, HbA1c, HOMA-IR, TyG based on Model 1.

### 4.5 Subgroup analysis

**Figure 3:**
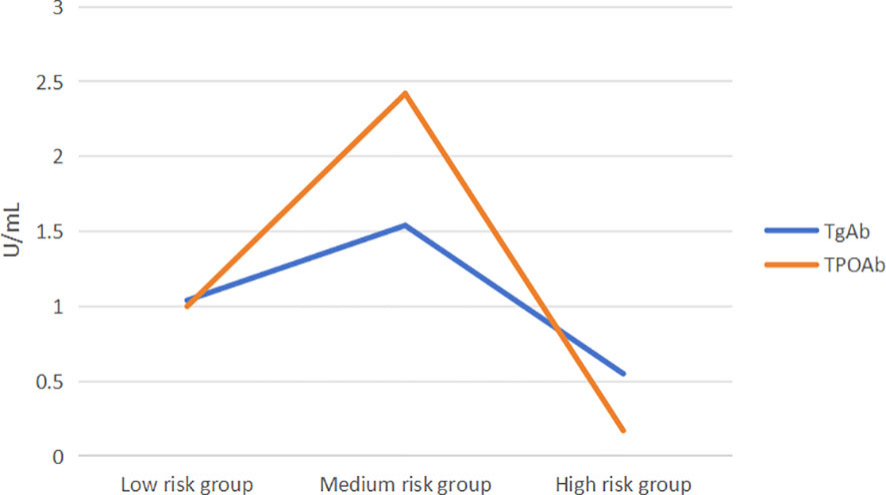
TgAb and TPOAb levels both exhibited an inverted “V” pattern.

Based on the FIB-4 index used to assess liver fibrosis, MAFLD patients were categorized into three subgroups: low-risk (FIB-4< 1.30), medium-risk (1.30 ≤ FIB-4 ≤ 2.67), and high-risk (FIB-4 > 2.67). As shown in **Table 5**, the medium-risk group had significantly higher levels of TgAb and TPOAb compared to both the low-risk and high-risk groups [TgAb: 1.04 (0.59, 2.83) vs 1.54 (0.76, 7.35) vs 0.55 (0.27, 1.32), *P*=0.035; TPOAb: 1.0 (0.29, 3.83) vs 2.42 (0.5, 23.08) vs 0.17 (0.09, 2.71), *P*=0.002].Further pairwise comparisons showed significant differences in TgAb levels between the medium-risk and high-risk groups (*P*=0.048), and significant differences in TPOAb levels between the low-risk and medium-risk groups, as well as between the medium-risk and high-risk groups (*P*=0.016, 0.014). Both TgAb and TPOAb levels displayed an inverted “V” pattern, indicating a peak in the medium-risk group (**Figure 3**).

**Table 5:** Comparison of thyroid function and antibody indicators among the three study groups.

Variables	Low-Risk Group (n=150)	Medium-Risk Group (n=100)	High-Risk Group (n=10)	χ2/t/H	*P*
FT3 (pmol/L)	4.16 ± 0.58	4.10 ± 0.64	4.14 ± 0.63	0.308	0.735
FT4 (pmol/L)	12.6 (11.58,13.74)	12.66 (11.35,14.2)	11.1 (10.31,12.79)	5.650	0.059
TSH (uIU/mL)	2.98 (1.76,4.13)	2.94 (1.97,4.0)	3.42 (3.05,4.98)	2.401	0.301
TgAb (U/mL)	1.04 (0.59,2.83)	1.54 (0.76,7.35)*	0.55 (0.27,1.32)	7.282	0.026
TPOAb (U/mL)	1.0 (0.29,3.83) **	2.42 (0.5,23.08)*	0.17 (0.09,2.71)	12.802	0.002
TgAb+	26 (17.33%)	30 (30%)	1 (10%)	6.028	0.041
TPOAb+	27 (18%)	29 (29%)	2 (20%)	4.175	0.12
TgAb/TPOAb+	38 (25.33%)	36 (36%)	3 (30%)	6.838	0.029

*P*< 0.05, statistically significant; * indicates a statistically significant difference between the medium-risk and high-risk groups, *P*-values adjusted using the Bonferroni correction; ** indicates a statistically significant difference between the low-risk and medium-risk groups,*P*-values adjusted using the Bonferroni correction.

## 5 Discussion

Our study found that T2DM patients with MAFLD exhibited higher levels of WBC, Lym, HsCRP, TgAb, and TPOAb, with a significantly increased prevalence of thyroid autoantibody positivity. This suggests that thyroid autoimmunity may contribute to MAFLD development, potentially through inflammatory pathways. Thyroid hormone variations are closely linked to metabolic syndrome, insulin resistance, obesity, and dyslipidemia (8). However, the literature on thyroid function and MAFLD association is inconsistent. Some studies report positive correlations between TSH, FT3, and MAFLD risk (10), while others, like Zuarth-Vázquez et al., found no such association (11).

Our study aligns with research indicating that elevated TSH levels significantly increase MAFLD risk, though TSH is not always an independent risk factor (12). Using the RCS model, FT4 levels were found to negatively correlate with MAFLD, with an inverted “V” pattern for FT3, indicating increased MAFLD risk with rising FT3 levels up to a certain point (13). Obesity-related studies show higher circulating FT3 levels (14), with Mendelian randomization linking higher BMI to increased FT3 through altered DIO expression and hypothalamic-pituitary-thyroid axis changes (15– 16).

Our findings indicate that within normal FT3 and FT4 ranges, these hormones are not associated with MAFLD risk. Importantly, we confirm that higher TSH levels significantly elevate MAFLD prevalence, reinforcing TSH as a risk factor for MAFLD in T2DM patients (17).

TgAb and TPOAb are the most common thyroid autoantibodies, closely associated with thyroid dysfunction, and frequently found in patients with autoimmune thyroid diseases. A 2018 cross-sectional study first suggested that thyroid autoimmunity might contribute to the pathogenesis of MAFLD through shared pathways (18). Research has shown that even within normal thyroid function, thyroid autoantibodies are closely linked to metabolic disorders (19). Evidence indicates that TPOAb and TgAb are associated with an increased risk of metabolic syndrome (20, 21). TPOAb is considered a risk factor for metabolic syndrome, while TgAb might reduce the risk of glucose and lipid metabolism disorders (22). However, another study found that the TyG index was significantly negatively correlated with FT4 and positively correlated with TSH and TgAb (23), highlighting a strong connection between thyroid dysfunction and insulin resistance. Our study did not find a relationship between the TyG index and TPOAb or TgAb. A five-year follow-up study showed that the risk of TSH abnormalities significantly increased in patients with persistently positive thyroid autoantibodies (24). As serum TSH levels increased, visceral obesity index and lipid accumulation product also increased significantly (25). Ye Hu et al. found that TSH and TPOAb levels were positively correlated with visceral and subcutaneous fat areas (26). Even after adjusting for TSH, the correlation with TPOAb remained, with TPOAb-positive patients having a fourfold increased risk of visceral obesity (26). These findings suggest that thyroid autoantibodies might be involved in MAFLD development, potentially causing metabolic dysfunction before thyroid dysfunction manifests.

Regarding glucose and lipid metabolism, our study did not find correlations with TgAb and TPOAb. Chronic inflammation is a known mechanism in MAFLD progression, and thyroid dysfunction may contribute to MAFLD through inflammatory pathways. A study on NASH showed that higher N/L ratios were associated with increased risks of NASH and fibrosis (27). The N/L ratio is closely linked to all stages of MAFLD, especially advanced inflammation and fibrosis (28, 29). However, Muammer Kara et al. found no correlation between N/L ratio and metabolic indicators or liver inflammation severity (30). In our study, T2DM patients with MAFLD had higher HsCRP and Lym levels and lower N/L and M/L ratios.

Elevated TPOAb and TgAb levels often indicate autoimmune thyroid disease, most commonly Hashimoto’s thyroiditis. Studies on euthyroid HT patients found higher NLR and PLR compared to healthy controls (31). Erhan Onalan et al. found correlations between PLR, NLR, TPOAb, TSH, and FT4, though no significant differences were observed between groups (32). PLR and NLR might indicate chronic inflammation progression, with insulin resistance and chronic inflammation potentially linking thyroid autoimmunity to MAFLD (33). Our study found significantly higher TgAb and TPOAb levels in T2DM patients with MAFLD. TPOAb was positively correlated with AST, Lym, and FINS, and negatively correlated with N/L and M/L; TgAb was positively correlated with AST and negatively with M/L. This suggests that inflammatory responses might play a crucial role in MAFLD development, potentially involving TgAb and TPOAb.

To further understand thyroid autoimmunity prevalence in MAFLD patients, we reviewed recent epidemiological studies. A Brazilian study found a cumulative incidence of TPOAb in euthyroid individuals of 1.46% over four years, with 1.23% in males and 1.67% in females (34). The Tehran Thyroid Study reported baseline TPOAb prevalence between 11.4% and 19.8%, with increasing trends among younger participants and decreasing trends among older ones (35, 36). Although female participants had higher TPOAb positivity rates, longitudinal trends were similar for both sexes. In China, the prevalence of TgAb and TPOAb positivity is 10.19% and 9.70%, respectively (37). Following the COVID-19 pandemic, TPOAb positivity significantly increased among survivors (15.7% vs. 7.7%), although 95% had normal TSH levels (38). Approximately one-quarter of MAFLD patients have hypothyroidism (39). Wang et al. found higher TPOAb positivity in T2DM patients with MAFLD compared to those with only T2DM (17% vs. 6.9%), especially among females (40). In our study, TgAb/TPOAb+ incidence was 11.4% in non-MAFLD patients and 29.6% in MAFLD patients, with 71.96% MAFLD prevalence in TgAb/TPOAb+ patients. Further analysis showed MAFLD prevalence increased with higher TPOAb and TgAb levels.

Few studies have explored the association between TgAb, TPOAb, and MAFLD. A 2020 survey found a negative correlation between TgAb and metabolic disorders, but only in single positive results (22). Xiaofu Zhang et al. found significant correlations between low TgAb, TPOAb, and high hsCRP in male MAFLD patients, while higher TgAb levels in females might be protective (12). Chenyi Wang et al. reported higher MAFLD prevalence in TgAb and TPOAb positive patients, with increased severe fatty liver cases at higher TPOAb titers. Hong Fan et al. suggested elevated TSH levels are closely associated with advanced liver fibrosis within normal thyroid function ranges (17). Our multivariate logistic regression analysis found that elevated TSH and TPOAb levels are risk factors for MAFLD, and ROC analysis suggested TPOAb and the TyG index might predict MAFLD prevalence. Elevated TPOAb is an important indicator for predicting autoimmune progression in T2DM patients with NAFLD, and higher TPOAb/TgAb levels might indicate more3severe MAFLD. The FIB - 4 (Fibrosis - 4) index is a non - invasive marker for liver fibrosis. It is calculated using age, aspartate aminotransferase (AST), platelet count, and alanine aminotransferase (ALT) (41). It helps in assessing the risk of liver fibrosis in patients. MAFLD patients categorized into low-risk (FIB-4<1.30, n=150), medium-risk (1.30 ≤ FIB-4 ≤ 2.67, n=100), and high-risk liver fibrosis groups (FIB-4 >2.67, n=10),showing an inverted “V” pattern of TgAb and TPOAb levels, indicating these levels might increase with early MAFLD fibrosis progression and decrease in advanced stages.

Our study shows that T2DM patients with MAFLD have significantly elevated TSH, TgAb, and TPOAb levels under normal thyroid function. Elevated TSH and TPOAb levels are risk factors for MAFLD in T2DM patients. TgAb and TPOAb levels vary among different liver fibrosis risk groups, showing an inverted “V” pattern. Under normal thyroid function, elevated TgAb and TPOAb levels might play an important role in MAFLD development and progression. There is a significant correlation between elevated TPOAb and MAFLD severity. As TPOAb titers increase, HbA1c, TC, TG, ALT, and TSH levels also rise, while FT4 decreases. Thyroid hormones influence glucose and lipid metabolism by regulating insulin secretion and sensitivity. Thyroid autoimmunity often leads to hypothyroidism, altering blood glucose and lipid levels in diabetes and NAFLD patients, worsening their condition. Immune system disruptions can lead to autoimmune diseases. Thyroid autoimmunity exacerbates immune imbalance, potentially contributing to diabetes and MAFLD (40).

Given the increasing clinical and economic burden of MAFLD globally, we aimed to analyze the etiological role of thyroid autoantibodies in MAFLD development. Some studies indicate significant associations between parathyroid hormone or thyroid function parameters (TSH, T4, T3, TPOAb) and MAFLD, while others do not confirm such relationships (42). The heterogeneity in study populations, sample sizes, diagnostic methods, and thyroid function assessments in each study complicates direct comparisons. If the association between thyroid autoantibodies and MAFLD is causal, it could open new opportunities for MAFLD treatment, screening, and prevention. Clinicians should consider monitoring TSH, TgAb, and TPOAb levels in T2DM patients, particularly those with normal thyroid function, to identify individuals at higher risk of developing MAFLD. Early detection of elevated thyroid autoantibody levels may prompt the initiation of lifestyle modifications or closer medical management. For patients with MAFLD, tracking changes in TgAb and TPOAb levels in the context of liver fibrosis risk could assist in assessing disease progression. This approach might guide decisions regarding more aggressive treatment or closer surveillance to prevent the development of advanced liver fibrosis. Additionally, further research could explore targeted therapies aimed at modulating thyroid autoantibody levels to potentially slow down or prevent the progression from MAFLD to liver fibrosis. We recommend extensive, long-term prospective studies to evaluate key thyroid function parameters and determine any causal links between thyroid diseases and MAFLD. Additionally, placebo-controlled randomized clinical trials should be conducted to determine whether thyroid autoantibodies or their analogs can effectively mitigate MAFLD progression and prevent its advancement. This study has several limitations. First, the cross-sectional design precludes the determination of causal relationships. Second, there may be potential selection bias or underrepresentation in the sample, affecting the generalizability of the findings. Third, confounding from unmeasured factors cannot be ruled out. Moreover, the assessment of liver fibrosis was relatively indirect, and there is a lack of long-term follow-up data on disease progression.

## 6 Conclusion

This study investigated the relationship between thyroid autoantibodies—thyroglobulin antibodies (TgAb) and thyroid peroxidase antibodies (TPOAb)—and metabolic dysfunction-associated fatty liver disease (MAFLD) in patients with type 2 diabetes mellitus (T2DM). Our findings demonstrate that elevated levels of thyroid-stimulating hormone (TSH), TgAb, and TPOAb are significant risk factors for MAFLD in T2DM patients, even when thyroid function is normal. The study reveals that these autoantibodies are associated with key metabolic indicators and inflammatory markers, highlighting their potential role in the pathogenesis of MAFLD. Furthermore, the data indicate an inverted “V”-shaped pattern in TgAb and TPOAb levels across different liver fibrosis risk groups, suggesting a dynamic interaction between thyroid autoimmunity and liver disease progression. This research underscores the importance of monitoring thyroid autoantibodies in T2DM patients as predictive markers for MAFLD, providing valuable insights for early detection, management, and the development of novel therapeutic strategies to combat this prevalent liver disease.

## Data Availability

The raw data supporting the conclusions of this article will be made available by the authors, without undue reservation.
